# A qualitative study of midlife women with type 2 diabetes in the UK: Exploring the impact of diabetes on their well-being

**DOI:** 10.1371/journal.pone.0350089

**Published:** 2026-06-01

**Authors:** Natalia Ioannou, Iliatha Papachristou Nadal

**Affiliations:** 1 Institute of Psychology, Psychiatry and Neurology, King’s College London, London United Kingdom; 2 Division for Care in Long Term Conditions, King’s College London, London, United Kingdom; University of Luzon, PHILIPPINES

## Abstract

**Background and aims:**

Type 2 Diabetes mellitus (T2DM) substantially influences the overall well-being and quality of life of individuals with diabetes and is widely associated with psychosocial burden. However, existing qualitative literature has largely focused on general adult populations, with limited attention to how illness experiences are shaped by gender and life-stage, particularly among midlife women in the UK. Moreover, the ways in which T2DM is integrated into identity, relationships, and future orientation during midlife remain insufficiently theorised. This study aimed to generate an in-depth, interpretive understanding of how midlife women with T2DM experience and make sense of the psychosocial, emotional, and self-management dimensions of the condition in their everyday lives.

**Methods:**

A qualitative study using online semi-structured interviews. Participants were recruited through a national social media campaign and an established research engagement database. Interviews were conducted with 13 midlife women with type 2 diabetes. Data were analysed using reflexive thematic analysis within an interpretivist framework.

**Results:**

The main four themes identified were Living with Type 2 Diabetes: encapsulates the practical aspects of living with diabetes, psychosocial dimensions: explores the broader impact of diabetes on participants’ lives, healthcare and support: sources for managing diabetes effectively and lastly outlook and personal development: captures participants’ perspectives on their treatment, their worries about the future, and the unexpected positive outcomes of living with diabetes. Together, these themes illustrate a dynamic process through which participants actively negotiate, reinterpret, and integrate T2DM within the context of midlife transitions, highlighting the interplay between illness, identity, and social roles.

**Conclusion:**

The findings highlight the need for a multifaceted approach to addressing the barriers and facilitators of diabetes care. This study extends existing qualitative literature by offering a life-stage- and gender-sensitive conceptualisation of living with T2DM, emphasising midlife as a critical period of identity renegotiation and future reorientation. By incorporating patients’ perspectives into care, they may enhance diabetes self-management and support more responsive, person-centred interventions, ultimately improving long-term well-being.

## Introduction

Diabetes Mellitus (DM) is a chronic metabolic disorder marked by ineffective insulin utilisation or inadequate insulin production [1]. Type 2 Diabetes Mellitus (T2DM) is the most prevalent, comprising about 90% of all DM cases [2]. In T2DM, elevated blood glucose levels persist due to both insulin resistance and deficiency [3]. The pathophysiology of T2DM involves both insulin deficiency and resistance [4].

In the United Kingdom, 4.7 million individuals have T2DM, projected to rise to 5.5 million by 2030 [5]. Globally, 573 million adults had diabetes in 2023, with numbers expected to reach 783 million by 2045 [2]. T2DM is a critical health concern and a primary cause of morbidity, disability, and mortality globally [6]. Diabetes affects 1 in 11 adults worldwide, making it the tenth most common cause of mortality [2,7].

Beyond its physical consequences, T2DM has substantial psychosocial implications, affecting emotional well-being, daily functioning, and overall quality of life [8,9]. Effective self-management, including dietary regulation and physical activity is crucial in mitigating disease progression [10,11].

### T2DM and emotional well-being

T2DM has a well-documented impact on psychological and emotional well-being [12]. Research demonstrates a correlation between diabetes and negative emotions such as anxiety, depression, and distress, which negatively affect clinical outcomes, including medication nonadherence and poor glycemic control [13,14].

Approximately one-third of individuals with Type 2 diabetes mellitus (T2DM) experience distress, often associated with difficulties in disease management, concerns about potential complications, and insufficient social support [13,15]. Social relationships may be strained by the demands of the condition; however, supportive networks can mitigate distress and improve overall well-being [16,17].

### T2DM and quality of life

Quality of Life (QoL) is significantly impacted by T2DM, as the condition often coexists with physical complications such as heart attack, stroke, and kidney failure, and mental complications including depression and anxiety. These factors collectively contribute to reduced well-being and increased healthcare burden [18].

Emerging evidence also highlights the role of positive psychological well-being in diabetes outcomes. A study of 3,907 individuals found that those with a stronger sense of purpose at the study’s start had a lower likelihood of developing pre-diabetes or T2DM, based on glycated hemoglobin (HbA1c) measurements. This finding is held after accounting for demographics, physical health, function, and psychiatric diagnoses [19]. Similarly, Boehm et al. [20] examined 7,800 participants over 13 years to explore the relationship between emotional vitality, optimism, and T2DM. Emotional vitality, but not optimism, was linked to a 9–15% reduction in self-reported T2DM diagnosis odds. This association remained significant even when accounting for clinical risk factors and demographics, though the effect size decreased when depressive symptoms were considered. Tsenkova et al. [21] further demonstrated that positive emotional states may reduce diabetes risk, particularly among individuals with a family history of the condition. Thus, early diagnosis and treatment of diabetes should primarily aim to improve QoL.

These findings suggest that psychological well-being is not only an outcome but also a potential protective factor in T2DM.

### Self-management

Effective self-management of T2DM is essential for reducing the risk of acute and chronic complications and improving overall well-being [22]. A thorough understanding of diabetes-related complications and psychosocial issues is crucial for successful interventions [23]. Illness perception influences patients’ adherence to prescribed medications [24]. Self-management education, which includes lifestyle modifications, medication knowledge, glucose monitoring, and coping strategies, is crucial for managing T2DM [25]. However, sustaining lifestyle changes is often challenging with social support, healthcare professional guidance, and motivation being critical factors for long-term success [26].

### Why this study focuses on women

This study focuses on midlife women, a group that may be particularly vulnerable to T2DM due to biological, psychological, and psychosocial factors. Women face higher risks, especially obesity and psychosocial stress, and experience unique physiological changes, such as menopause, which may influence metabolic function and heighten their susceptibility to diabetes [27].

These physiological changes, combined with socio-economic factors such as education and income, increase the likelihood of T2DM in women [28]. Women also experience more distress and stigma related to diabetes, which negatively impacts their QoL [29].

In addition, women with T2DM experience higher cardiovascular risks and increased mortality from heart disease and stroke further complicate diabetes management in women [30]. These gender differences highlight the need for more targeted and context-sensitive approaches to diabetes care. Research highlights the need for better management strategies for women, including addressing mental health and improving healthcare communication [31].

In this context the present study adopts a qualitative approach to explore how midlife women experience and interpret the impact of T2DM on their well-being across different cultures. By focusing on lived experience this study is expected to offer evidence-based recommendations for healthcare services, enhancing diabetes management for midlife women in diverse cultural settings. The results aim to guide healthcare providers and policymakers in addressing the unique challenges faced by this population in managing T2DM.

## Methods

### Research design and methodology

This study employed qualitative methodology through online semi-structured interviews to investigate midlife women’s perspectives on diabetes and its influence on well-being. The study was situated within an interpretivist qualitative paradigm, which assumes that reality is socially constructed and that meaning is co-produced through interaction between researcher and participant [32]. The methodological approach adopted was reflexive thematic analysis, as developed and updated by Braun and Clarke [33], which is theoretically flexible but grounded in an interpretivist epistemological position. This paradigm aligns with reflexive thematic analysis, which emphasises subjectivity, interpretation, and the researcher’s active role in knowledge production.

Participants were recruited using a pre-existing research engagement database developed through a prior national social media recruitment campaign. The database comprised approximately 600 women with diabetes who had previously expressed interest in research participation. This established sampling frame enabled focused identification of eligible participants, supporting efficient and purposeful recruitment within the qualitative paradigm. In qualitative research, rigour is determined by the richness and relevance of data and analytic depth rather than the absolute duration of fieldwork [33,34].

Interviews were recorded and transcribed using Microsoft Teams, then coded and analysed by NI using NVivo (Version 12, QSR). Each transcript was reviewed for accuracy before analysis. Thematic analysis was employed to recognise, analyse, and describe patterns within the data, allowing a deeper understanding of participants’ experiences, with reflexive thematic analysis guiding an interpretive and flexible engagement with the data [33].

The study design, data collection, and reporting were informed by the Consolidated Criteria for Reporting Qualitative Research (COREQ) checklist (S1 Appendix), and the structure of this section has been aligned with COREQ domains to enhance transparency, rigour, and methodological clarity.

### Participant recruitment and eligibility

A recruitment approach was conducted from 25th March to 8th April 2024 using a multi-channel strategy, including a social media campaign on Google, Facebook, and the last author’s X (Twitter) account (IPN). Recruitment was conducted through a national social media campaign designed to enable participation from individuals residing in different regions of the UK. The term **“**UK-wide ” is used to describe the geographical reach of recruitment rather than to imply statistical or demographic representativeness. Consistent with qualitative research methodology, the study did not aim to produce a sample representative of the UK population but to obtain rich, in-depth accounts of lived experience. Purposeful sampling strategies prioritised experiential relevance and variation over demographic proportionality [35,36].

A total of 60 women responded to the campaign. After screening for eligibility based on the study’s inclusion criteria, 13 women were purposively selected and formally included as participants. This strategic recruitment process ensured that participants had relevant lived experience and insight to contribute meaningfully to the study aims. It also enhanced the trustworthiness and rigour of the dataset. Sample adequacy was guided by the richness and relevance of the data rather than traditional data saturation, consistent with the principles of reflexive thematic analysis [33,34,37].

Prospective participants completed a pre-screening Microsoft Form with demographic details and verification of T2DM and provided an email address for further communication. Selected participants were contacted via email and received a £20 High Street voucher. This process effectively combined recruitment and early data collection.

The study was conducted entirely online to enable inclusion of women from different regions and backgrounds without geographical or mobility constraints. Recruitment advertisements were shared across multiple platforms and diabetes-related forums to reach individuals of varying ages, ethnicities, and socioeconomic backgrounds. Study materials and interviews were conducted in English; therefore, participants were required to be fluent in English to ensure clarity and analytic consistency. Although this may have limited inclusion of non-English speakers, the use of a single interview language is methodologically common in qualitative research practice. Multilingual data collection requires extensive translation and interpretive procedures, which introduce additional methodological and conceptual challenges, particularly in preserving meaning across languages and was therefore beyond the scope of the present study [38,39].

### Participants: Inclusion and exclusion criteria

The sample size consisted of 13 participants. Sample adequacy was considered in relation to the depth, specificity, and quality of the data, consistent with the concept of information power, rather than the achievement of data saturation [34].

The inclusion criteria were women aged 45–65 years, selected to represent midlife, a developmental period characterised by significant biological and psychosocial transitions, including the menopausal transition and early post‑menopause [40–42]. Midlife is widely recognised as a pivotal stage for women’s health, involving hormonal changes, shifts in metabolic function, and evolving social and health roles, all of which are associated with increased vulnerability to type 2 diabetes and may shape illness experiences and self‑management.

Participants were required to have been diagnosed with T2DM for at least two years to ensure sufficient lived experience of diagnosis, treatment initiation, and ongoing self‑management. Two participants reported a shorter duration since diagnosis (approximately one year); however, they were retained in the study due to the relevance and richness of their accounts. Their inclusion was considered to enhance the interpretive depth of the dataset by capturing experiences from earlier stages of adjustment. In qualitative research, sampling decisions are often guided by the richness, relevance, and diversity of experiences rather than strict eligibility thresholds [33,43].

Additionally, participants were required to be fluent in English, as interviews were conducted in English and translation resources were not available. Although some participants came from diverse cultural backgrounds, the interview questions were designed to be culturally neutral and sensitive, allowing participants to share their experiences freely.

Exclusion criteria included individuals with mental health issues such as depression, anxiety, or severe mental illness [44], minors, prisoners, and those unable to provide informed consent. This exclusion criterion was applied to minimise potential distress during interviews; however, it may limit the inclusion of perspectives where psychological comorbidity is present, and this is acknowledged as a limitation. No restrictions were placed on ethnicity or socioeconomic background, as the study aimed to explore experiences of women with T2DM across diverse backgrounds.

To justify the adequacy of the sample, data collection and analysis were conducted concurrently, allowing ongoing assessment of the richness and relevance of the data. In line with reflexive thematic analysis, sample size was guided by the concept of information power, whereby the specificity of the study aim, the relevance of participants’ experiences, and the depth of the interviews supported analytic sufficiency. Data collection continued until the dataset provided sufficient conceptual richness, with later interviews contributing to the refinement and elaboration of emerging themes rather than generating entirely new codes [34].

Participant characteristics are presented in Table 1.

**Table 1:** Participant characteristics (N = 13).

Participant ID	Age	Ethnicity	Duration of diagnosis
P1	51	European	3 years
P2	56	White English	11 years
P3	51	Indian	3 years
P4	54	Mixed White and Asian	13 years
P5	58	White British	34 years
P6	56	White British	5 years
P7	57	White British	1 year
P8	63	Welsh	23 years
P9	62	Irish	22 years
P10	45	White British	23 years
P11	61	Chinese and English	1 year
P12	64	White British	2 years
P13	47	Mixed White and Asian	3 years

### Interview procedure and material

Prior to participation, individuals were required to sign a written informed consent form and were provided with an information sheet outlining the purpose, procedures, potential benefits, and limitations of the research. Interviews were conducted remotely through the Microsoft Teams platform, following a briefing session to clarify the process and address any participant questions. Each interview lasted approximately 40 minutes and included open-ended questions designed to elicit detailed and reflective responses (S2 Appendix: Interview Schedule and Topic Guide). The use of videoconferencing for qualitative data collection is supported by previous research, highlighting its feasibility, acceptability, and capacity to facilitate in-depth qualitative interviews [45,46].

### Development of the interview guide

The semi-structured interview guide was developed through a systematic, six-step process grounded in existing theory and literature. First, the study objectives were clarified, focusing on midlife women’s experiences of Type 2 Diabetes Mellitus (T2DM), including psychosocial aspects such as distress, stigma, self-management, and potential cultural influences. Second, an extensive literature review was undertaken to identify recurrent themes in qualitative research on diabetes-related stigma, emotional adjustment, and coping [47]. The Health Belief Model and psychosocial adjustment theories informed the conceptual structure of the guide. Third, an initial pool of questions was generated based on these themes, covering key domains such as diagnosis experiences, emotional responses, lifestyle changes, social relationships, and interactions with healthcare services. Fourth, the research team (NI and IPN) reviewed the draft guide to ensure clarity, relevance, and alignment with the study aims. Feedback led to refinements in language, logical sequencing, and the sensitivity of specific questions. Fifth, the revised guide was pilot tested with one patient and public involvement (PPI) participant living with T2DM. Feedback from this pilot interview led to further adjustments, including refining phrasing and improving transitions between topics to support a natural conversational flow. Finally, the guide was fully refined and finalised.

The completed interview guide consisted of four thematic sections:

1. Diagnosis and early experiences with T2DM
2. Lifestyle changes and daily management
3. Psychosocial and emotional aspects of living with diabetes
4. Healthcare experiences and sources of support

This structure ensured coverage of key psychosocial domains while allowing flexibility for participants to elaborate on personal experiences.

### Interview Guide

A semi-structured interview guide was used to explore participants’ experiences of living with type 2 diabetes as women within their cultural communities. The guide included questions on demographic characteristics (such as age and ethnicity), diabetes history (including age at diagnosis and presence of symptoms), and perceptions of wellbeing. It also examined participants’ experiences of lifestyle changes, emotional wellbeing, healthcare interactions, social support, cultural expectations, and stigma. Additional prompts addressed coping strategies, sources of information, the role of social media, and culturally specific attitudes toward medical conditions and medication. The guide was designed to be flexible, allowing participants to elaborate on areas most relevant to their experiences. The full interview guide is provided in the S3 Appendix.

### Data collection

Semi-structured interviews were conducted and recorded for accurate transcription remotely using Teams, guided by questions on emotions associated with diabetes, impact on daily life, attitudes of healthcare professionals, and coping mechanisms. The first author (NI), a female researcher with a background in psychology and MSc training in Mental Health Studies, conducted all interviews under the supervision of an experienced PhD-level qualitative researcher (IPN). Interviews were conducted between 22 April and June 2024. Brief field notes were taken after the interviews, mostly reflecting on the participant’s engagement (talkative or reserved). These notes were used to support reflexive engagement and to enhance contextual understanding during the research process. While they informed the researcher’s interpretative lens, they were not directly coded or included as part of the formal dataset, which consisted of the interview transcripts.

### Interview approach

Interviews followed a logical sequence, transitioning from general to specific questions, using sensitive and appropriate language for participants. The researcher (NI) contacted participants prior to the interview to arrange scheduling, which also facilitated initial rapport-building before the interview commenced. No prior relationship existed between the researcher and participants before recruitment, beyond initial contact for scheduling. Participants were informed that the interviewer was a female researcher with a background in psychology, currently undertaking an MSc in Mental Health Studies, with a research interest in diabetes and women’s health.

### Data analysis

Ιnterviews were audio-recorded, transcribed verbatim, and stored electronically. Data were analysed using reflexive thematic analysis [33], which emphasises the active role of the researcher in interpreting and constructing themes, rather than seeking coding reliability or consensus. Analysis followed the six phases described by Braun and Clarke [48], informed by their later work on reflexive thematic analysis and quality practice [33]: (1) familiarisation with the data; (2) generating initial codes; (3) developing themes; (4) reviewing themes; (5) defining and naming themes; and (6) producing the final report. Table 2 illustrates the application of these six phases, outlining the key analytic steps, outputs, and activities at each stage.

**Table 2:** Application of reflexive thematic analysis (adapted from Braun & Clarke [33,48]).

Phase	Key activities	Analytic output
1. Familiarisation with data	Repeated reading of transcripts, note-taking	Initial observations and ideas
2. Generating initial codes	Inductive line-by-line coding in NVivo	List of initial codes
3. Developing themes	Grouping codes into potential themes	Preliminary theme structure
4. Reviewing themes	Comparing themes to dataset for coherence	Refined themes and subthemes
5. Defining and naming themes	Reflexive discussion, conceptual clarity	Final theme definitions
6. Producing the report	Selection of illustrative quotes, writing results	Completed thematic narrative

An inductive coding approach was employed, deriving codes and themes directly from participants’ accounts. The analytic process was iterative and recursive, moving back and forth between data, codes, and themes.

Both researchers (NI and IPN) first read all transcripts to gain familiarity with the dataset. NI conducted initial coding as an interpretive and reflexive process, while IPN provided ongoing supervision, critical reflection, and analytical guidance. Discussions between researchers explored alternative interpretations and enhanced analytic depth, without seeking coding consensus or inter-rater reliability, consistent with reflexive thematic analysis principles.

NVivo 12 supported organisation, coding, and management of the data, while reflexive memos documented analytic decisions, ensuring a transparent audit trail. Data were pseudonymised after analysis. Data were pseudonymised after analysis.

To enhance transparency and provide a clear audit trail, the analytic process involved several iterative steps. First, transcripts were read repeatedly to support deep familiarisation and initial notetaking. Second, initial codes were generated inductively across the dataset, capturing both semantic and latent meanings. Third, codes were grouped into preliminary themes based on shared meaning. Fourth, themes were reviewed and refined through comparison with the dataset. Fifth, themes were defined and named through reflexive discussion, focusing on conceptual clarity and relevance. Throughout this process, reflexive memos documented analytic decisions and shifts in interpretation.

To further enhance transparency, the development of codes into themes was systematically documented. Table 3 presents the final themes and subthemes derived from inductive coding, alongside the associated codes (nodes) and illustrative quotes from participants, providing a clear visual account of how the data were interpreted and organised.

**Table 3:** Themes, subthemes, codes, and illustrative quotes from reflexive thematic analysis.

Theme	Subtheme	Example codes (nodes)	Illustrative quote
Living with Type 2 Diabetes	Diagnosis and Health History	Diagnosis; Health, History	“I was diagnosed about two years ago…”
	Symptoms and Effects	Symptoms; Physical Effects	“My symptoms were quite severe before”
	Management Strategies	Management; Lifestyle Changes	“I also go for complementary therapies…”
Psychological Dimensions	Psychological and Emotional Impact	Psychological impact; Emotional Responses	“Sometimes I give in to emotions…”
	Social and Cultural Aspects	Social Influence; Cultural Beliefs	“It’s affected my social life…”
	Daily Life Impact	Routine Changes; Lifestyle Disruption	“It’s changed my lifestyle…”
Healthcare and Support	Experiences with Healthcare System	Healthcare Experiences	“My experience of the NHS is very positive”
	Information and Support Sources	Information Sources; Online Support	“I found the website diabetes.co.uk helpful”
	Challenges in Diabetes Management	Challenges; Barriers	The challenges is keeping it up”
Outlook and Personal Development	Attitudes Towards Treatment	Treatment Attitudes	“I don’t want to be on medication…”
	Long-term Concerns	Future Worries; Complications	“It affects everything…stroke, heart attack…”
	Positive Aspects and Opportunities	Positive Reframing	“It’s made me think about my wellbeing”
	Personal Growth and Self-awareness	Personal Growth; Awareness	“I’m aware of portion control…”

Theme development was guided by conceptual relevance and interpretive depth rather than frequency, with no quantitative representation used to determine importance.

### Ensuring rigour and trustworthiness

To ensure the rigour and trustworthiness of this qualitative study, we adopted multiple strategies aligned with reflexive qualitative research principles. Methodological coherence was maintained by ensuring alignment between the interpretivist paradigm, the qualitative design, and the reflexive thematic analysis approach.

Credibility was enhanced through a systematic and iterative analytic process. Both authors engaged in repeated readings of the transcripts and ongoing reflexive discussions of theme development and interpretation, ensuring close engagement with the data.

Dependability was supported by maintaining an audit trail documenting coding, theme development, and reflexive analytic decisions throughout the research process. NVivo 12 software, alongside the use of memos and annotations, facilitated systematic organisation of the data and supported transparency from initial coding to final theme development.

Confirmability was strengthened through reflexivity. The primary researcher kept reflexive notes throughout data collection and analysis, documenting assumptions, analytic choices, and potential influences on interpretation. Regular reflexive dialogue between the researchers supported critical examination of interpretations and challenged initial assumptions.

Lastly, transferability was facilitated by providing rich, detailed descriptions of the study context and participant characteristics, supported by illustrative participant quotations in the findings.

### Ethical considerations

Ethical approval was obtained from King’s College London Research Ethics Office (LRU/DP-23/24–40835), on March 4, 2024. Written consent was secured from all participants before the data collection process starts. The data were securely anonymised and stored on King’s College London’s OneDrive platform. All participants provided written informed consent for the use of anonymised data in publications, and no potentially identifying details are included in the manuscript. As recruitment occurred across the UK and only broad demographic information is reported, individuals cannot be identified.

## Results

### Demographics

A total of 13 women aged between 45 and 64 years participated in the study. All participants identified as female. Most participants were identified as White British, with while a smaller number represented diverse ethnic backgrounds, including Indian, European, and mixed ethnicities. The duration of Τ2DM diagnosis varied considerably, ranging from 1 to 34 years, reflecting a heterogeneous sample in terms of illness experience. No drop-out or non-participation was recorded during the study.

## Qualitative results

The analysis generated four overarching themes that capture participants’ experiences of living with type 2 diabetes: (1) Living with Type 2 Diabetes, (2) Psychosocial Dimensions, (3) Healthcare and Support, (4) Outlook and Personal Development.

These themes reflect both the practical and experiential dimensions of diabetes, encompassing how participants understood their diagnosis, managed their condition, and interpreted its impact on their everyday lives.

### Theme I: Living with Type 2 diabetes

This theme reflects how participants interpreted and navigated diabetes as an ongoing condition embedded in everyday life, encompassing diagnosis, bodily experiences, and self-management practices. Rather than representing solely medical aspects, participants’ accounts highlighted how living with diabetes involved continuous negotiation and adaptation. This suggests that diabetes was not experienced as a static diagnosis but as an evolving condition requiring ongoing identity work and reinterpretation of the self in relation to health and illness. Participants’ accounts highlighted how diabetes was experienced as an ongoing condition embedded in everyday life, shaping daily routines, symptom monitoring, and management strategies.

#### Diagnosis and health history.

Participants described diagnosis as occurring across different life stages, ranging from recent to long-standing experiences, shaping their understanding of and response to the condition over time.

Some participants recalled diagnosis as a supported and structured process, particularly when healthcare professionals played an active role:

*“ […] I was diagnosed about two years ago— and I had an excellent GP who was very helpful”, “I was diagnosed 11 years ago*” (P2).

Others described diagnosis as unexpected and incidental, often identified during investigations for unrelated conditions:

“*[…] Because I was in the hospital for COVID, they took my blood, and my HBA1c was really high*” (P13).

These accounts illustrate that diagnosis was not always accompanied by clear symptoms, but rather emerged through routine or acute healthcare encounters, shaping participants’ initial sense-making of the condition.

#### Symptoms and effects.

Participant’s accounts revealed that diabetes was experienced through a combination of perceived symptoms, ongoing self-monitoring, and anticipation of potential complications.

Some participants described intense or disruptive bodily symptoms, particularly prior to diagnosis:

*“[…] My symptoms were quite severe before. I looked into them*” (P3).

At the same time, ongoing self-monitoring appeared central to participants’ sense of agency and reassurance:

*“[…] I have my tests. My eyes are fine. My feet are fine. I can feel sensations*” (P13).

References to complications- such as vision problems, cardiovascular risk, and concerns about limb health- highlighted how living with diabetes extended beyond present symptoms to include awareness of future health risks:

“*[…] Sometimes I get blurry vision*” (P2), “*Stroke, heart attack, kidney issues— it affects everything”*. “*I’m obsessed with keeping my feet healthy because I don’t want to lose any toes or feet*” (P5), “*I’ve got a mark on my shin*” (P7).

These narratives demonstrate that living with diabetes involved both current bodily experiences and an ongoing vigilance toward potential deterioration. This reflects how participants engaged not only with present symptoms but also with anticipated futures, where awareness of potential complications shaped ongoing monitoring, decision-making, and perceptions of vulnerability.

#### Management strategies.

Participants described actively engaging in a range of management strategies, reflecting sense of responsibility, agency, and personal investment in controlling their condition.

Some accounts challenged dominant perceptions of diabetes as inevitably progressive, instead framing it as a condition that could be stabilised or improved through active engagement:

“*[…] People think it can’t be managed well, or it can’t be put into remission*” (P13), “*And I’m virtually in remission now after a year*” (P11).

Management strategies included both biomedical and self-directed approaches, including medication adherence, lifestyle modification, and complementary practices:

“*[…] Along with medication and medical support, I also use complementary therapies*” (P13).

In several cases, participants described seeking additional expertise or support to manage symptoms and improve wellbeing, highlighting the complex, evolving nature of self-management:

“*[…] I had to employ a nutritionist to help me understand and manage my exhaustion, which took about 12 months*” (P7).

These accounts suggest that managing diabetes was not a linear or purely medical process, but rather a dynamic practice requiring ongoing adaptation, knowledge-seeking, and interpretive work. Beyond these practical and bodily experiences, participants’ accounts also revealed the broader psychosocial and emotional dimensions of living with diabetes, explored in the next theme.

### Theme II: Psychosocial dimensions

This theme captures how living with type 2 diabetes extended beyond physical management to shape participants’ emotional well-being, social identities, and everyday interactions. Participants’ accounts highlighted that diabetes was experienced as a personally meaningful and socially embedded condition, influencing relationships, cultural practices, and daily routines. Rather than being purely in a medical condition, it was intertwined with identity, social participation, and emotional life.

#### Psychological and emotional impact.

Participant’s narratives revealed a dynamic, interpretive interplay between emotional states and diabetes management, where emotions were both shaped by and actively shaping self-care behaviours.

Some participants described emotion-driven decision-making, particularly in moments of vulnerability:

“*[…] Sometimes I give in to emotions, like having something unhealthy on a bad day, I will have something that maybe isn’t healthy* “ (P13).

Additionally, diabetes was frequently constructed as an emotionally burdensome condition, associated with distress, pressure, and a perceived loss of balance across different life domains:

“*[…] I feel poor in all areas of my life—mental health, physical health, and well-being—and high stress levels have worsened my diabetes significantly*” (P4).

Given participant’s narratives diabetes management was experienced not merely as a set of behaviours or medical routines, but as an emotionally embedded process in which stress, frustration, and self-regulation were closely intertwined. Participants’ reflections suggest that emotional experiences were integral to how they interpreted, prioritised, and enacted self-care practices. These findings indicate that emotional responses were not merely consequences of the condition but actively shaped how participants engaged with self-management, highlighting a dynamic and bidirectional relationship between emotion and health behaviour.

#### Social and cultural aspects.

Participants described diabetes as reshaping their social participation and relational dynamics, particularly through food-related practices and shared routines.

Dietary restrictions often required negotiation and adaptation in social contexts, sometimes leading to feelings of exclusion or disruption:

“*[…] It’s affected my social life because I have to say no to some things or avoid events with alcohol and sugar*” (P13),

Changes in eating practices also influenced intimate relationships, highlighting how diabetes extended into domestic life:

“*[…] It affected mealtimes—I was eating differently from my husband, impacting our relationship*” (P3).

Cultural meanings and personal narratives around diabetes further shaped how participants positioned themselves in relation to the condition. Some participants appeared to reframe or soften the illness identity, potentially to maintain a sense of normality or control:

“*[…] I prefer to say I am eating to support my metabolism rather than saying I have type 2 diabetes*” (P13).

These narratives illustrate that diabetes was socially negotiated, culturally interpreted, and relationally mediated, with participants actively constructing a sense of identity and control alongside disease management. In this way, participants appeared to negotiate their social roles and identities in relation to diabetes, balancing expectations of normality, femininity, and cultural practices with the demands of disease management.

#### Daily life impact.

Participants identified diabetes as an ongoing, pervasive influence on daily routines, requiring continual adaptation across work, leisure, and self-care domains.

For some, the condition had substantial disruptive consequences, particularly regarding employment and functional capacity:

“*[…] I’ve had to give up work due to symptoms and complications*” (P10).

For others, diabetes prompted intentional lifestyle modifications, often framed as positive or necessary adjustments to well-being:

“*[…] It’s changed my lifestyle by making me more careful about what I eat, portion control, and choosing unprocessed foods*” (P13).

In several cases, these changes were described as permanent or long-term adaptations, reflecting a reconfiguration of habits, priorities, and health-oriented behaviours:

“*[…] The change in lifestyle was permanent to address the issue*” (P12).

Taken together, these experiences suggest that diabetes functioned simultaneously as a constraint and a catalyst, limiting certain aspects of daily life while encouraging reflection, self-regulation, and new forms of health-oriented behaviour. This interpretive perspective emphasises how participants negotiated meaning, identity, and agency in relation to living with diabetes. This reflects an ongoing tension between disruption and adaptation, where participants sought to maintain a sense of normality while incorporating the demands of chronic illness into everyday life.

### Theme III: Healthcare and support

This theme explores how participants navigated healthcare systems, accessed information, and mobilised support in managing T2DM. Experiences of care were not uniform but shaped by access, communication, and individual expectations, often requiring participants to take an active role in coordinating their own support.

#### Experience with healthcare system.

Participants reported described diverse and sometimes contrasting experiences with healthcare services, reflecting variability in access, communication, and perceived quality of care.

Some accounts reflected dissatisfaction and perceived lack of support, suggesting gaps in care provision:

“*[…] Extremely poor, next to no help or care*” (P8).

In contrast, others described positive and supportive relationships with healthcare professionals, particularly when care was accessible and responsive:

“*[…] My experience with the NHS in Kent is very positive*” (P12). “*The diabetic nurses are very good- I can ask them anything- I’m well looked after*” (P9).

It seems that experiences of care varied considerably, indicating that engagement with healthcare services was contingent on the quality of interaction and continuity of support. Where relationships were perceived as supportive, participants expressed greater confidence in managing their condition, whereas limited or inconsistent care appeared to shift responsibility more heavily onto the individual. This was often described as a fragmented experience, where continuity and clarity were not always guaranteed, requiring participants to actively navigate services and interpret guidance rather than passively receive care.

#### Information and support sources.

Participants drew on a range of information sources, often combining formal, informal, and self-directed resources to support diabetes management.

Online platforms emerged as important sources of accessible and ongoing information, with some participants highlighting specific websites:

“*[…] I find the website diabetes.co.uk very helpful and balanced*” (P12).

At the same time, participants recognised the limitations of formal organisations, particularly in relation to personalised advice:

“*[…] They wouldn’t give individual advice, which is understandable*” (P8).

However, the broader use of digital resources, including online articles and video content, further illustrates the role of self-directed learning in everyday management:

“*[…] Online articles and YouTube videos*” (P13).

Family support was described as a key enabling factor in sustaining lifestyle changes and emotional well-being:

“*[…] My husband’s support is very important-We cook meals from scratch and monitor sugars*” (P2), “*I have very good family support*” (P12).

However, not all participants had access to such support, and some described managing diabetes as an individual and sometimes isolating experience:

“*[…] The most important source of support is myself*” (P8), “*It’s been quite lonely; I have to rely on myself for information*” (P3).

Based on participants’ narratives, it appears that the availability of support varied, with some participants drawing on strong relational networks, while others navigated diabetes largely through self-management and independent information-seeking. This variability highlights how support was not only accessed but actively constructed, often reflecting broader social and relational contexts.

#### Challenges in diabetes management.

According to participants, managing diabetes is an ongoing and demanding process, requiring sustained effort across multiple aspects of daily life.

Some accounts reflected difficulties in maintaining overall well-being, with emotional and physical health perceived as interconnected:

“*[…] I think well-being is very important, and I don’t have any well-being. So, that’s probably why my Type 2 diabetes is out of control*” (P4).

Sustaining long-term behavioural changes emerged as a particular challenge, highlighting the ongoing nature of self-management:

“*[…] The challenge now is keeping it up—the change in lifestyle must be permanent*” (P12).

Participants also described specific challenges related to diet, exercise, and stress, suggesting that management extended beyond isolated behaviours to broader life circumstances:

“*[…] My recent results were not great because I’m stressed. Maybe I am not doing enough exercise? It’s not just about struggling with my diet*” (P1).

Managing diabetes emerged as a complex and evolving process, shaped not only by individual behaviours but also by emotional states, competing life demands, and the sustainability of lifestyle changes over time. These accounts underscore how responsibility for management was often individualised, requiring ongoing effort, adaptation, and self-regulation in the context of everyday pressures. Participants’ experiences highlighted a continual negotiation between formal healthcare provision and self-directed practices, with individuals actively integrating, adapting, and sustaining care strategies within the realities of their daily lives.

### Theme IV: Outlook and personal development

This theme reflects how participants interpreted their future with diabetes, negotiated treatment decisions, and, in some cases, redefined their sense of self through processes of adaptation and personal growth. Rather than viewing diabetes solely as a burden, participants’ accounts revealed a more complex interplay between concern, agency, and transformation. This highlights how living with diabetes was also oriented toward the future, shaping expectations, decisions, and evolving identities.

#### Attitudes towards treatment.

Participant’s attitudes towards treatment reflected an ongoing negotiation between medical recommendations and personal beliefs about health, control, and autonomy.

Many participants expressed a preference for lifestyle-based management, positioning it as a more desirable or empowering alternative to long-term medication use:

“*[…] I want to achieve good health for myself. I have the motivation because I don’t want to be on medication for the rest of my life*” (P13).

However, medication was also recognised as necessary in certain circumstances, illustrating a pragmatic and context-dependent engagement with treatment:

“*[…] Medication was the only solution sometimes*” (P3).

Cultural beliefs and personal values appeared to shape treatment decisions, particularly in relation to resistance towards pharmacological intervention:

“*[…] I didn’t want to go on medication. The doctors just wanted to put me on medication. I tried to address it through diet and exercise*” (P3).

Concerns about side effects, further complicated treatment choices, contributing to hesitancy or ambivalence:

“*[…] I don’t want to take more insulin because it will cause weight gain. I don’t want to take some painkillers due to side effects*” (P8).

A perceived lack of clear communication from healthcare professionals sometimes intensified uncertainty and shifted responsibility onto participants, requiring them to independently evaluate treatment options:

“*[…] Extremely poor care, and I had to make my own suggestions—I loathe looking on Google for medication because that’s no way to do anything*” (P8).

These accounts demonstrate that treatment decisions were actively negotiated rather than passively accepted, shaped by trust, knowledge, and efforts to maintain control over the body and future health.

#### Long-term concerns.

Participants’ reflections on the future revealed ongoing uncertainty and concern regarding the long-term implications of diabetes.

For some, the condition prompted heightened awareness of overall health and future risks:

“*[…]* It has made me think much more about my overall well-being” (P12).

Others described more profound and emotionally charged concerns, particularly where diabetes intersected with life aspirations and long-term identity:

“*[…] I had insulin resistance, which continued through adulthood. It’s devastating because I’ve always wanted to be a mom. Now, I have multiple complications from Type 2 diabetes. It’s a daily battle to keep the weight down*” (P10).

From participants’ accounts, diabetes was experienced not only as a present condition but also as a trajectory influencing expectations, future possibilities, and life planning. In this way, the condition extended into imagined futures, influencing how participants understood their life course and personal aspirations.

#### Positive aspects and opportunities.

Despite the challenges, some participants identified positive dimensions of living with diabetes, reframing the condition as an opportunity for improved health awareness and behavioural change.

For certain individuals, diabetes was integrated into everyday life in a pragmatic and manageable way:

“*[…] For me, it’s not a challenge. I just get on with my day-to-day living and do the best I can for my health*” (P9).

Others described a shift towards a more holistic understanding of health, extending beyond glycemic control:

“*[…] It’s about being fit, taking time to relax, and not just worrying about diet and blood sugar. Diabetes has made me think much more about my overall well-being*” (P12).

These accounts suggest that diabetes could also be reinterpreted as a catalyst for reflection, enabling participants to re-evaluate priorities and adopt broader understandings of well-being.

#### Personal growth and self-awareness.

Participants’ accounts highlighted processes of learning, adaptation, and increased self-awareness, often emerging in response to gaps in formal support.

In the absence of sufficient guidance, some participants engaged in extensive self-directed learning, taking responsibility for understanding and managing their condition:

“*[…] There wasn’t much support, so I had to spend a lot of time researching and learning about what to do, like rapid weight loss*” (P3).

This active engagement appeared to foster greater awareness of bodily processes, dietary practices, and health behaviours:

“*[…] I’m aware of portion control, processed food, and the glycemic index*” (P13).

For some, diabetes was framed as a turning point or “wake-up call”, prompting lasting lifestyle changes:

“*[…] It’s a warning call that you need to change how you live and what you eat*” (P12).

Collectively, these accounts highlight that living with diabetes prompted ongoing reflection and negotiation of personal priorities, with participants actively redefining what health and well-being meant for them. This theme illustrates how engagement with T2DM extended beyond immediate management to encompass identity reconstruction, future-oriented planning, and the development of self-efficacy. Participants described how challenges and successes shaped their sense of agency, enabling them to interpret the disease not merely as a constraint, but as a catalyst for growth, learning, and a reorientation of life goals. In doing so, they demonstrated an emergent form of health literacy and resilience, where personal experience and self-directed strategies informed ongoing decision-making and adaptation.

## Discussion

This study provides an interpretative account of how midlife women with type 2 diabetes mellitus (T2DM) understand and manage their condition within the context of their everyday lives. Moving beyond predominantly behavioural models of self-management, the findings demonstrate that living with T2DM is an ongoing process of negotiation and meaning-making, in which women actively interpret their diagnosis, adapt management practices, and integrate the condition into their identities, social roles, and future expectations. Importantly, these processes are situated within a framework of humanised care, in which empathy, relational support, and recognition of patients as whole persons shape both engagement and interpretation of health practices [49].

Synthesising across themes, the findings suggest that living with T2DM is not experienced as a discrete or static condition but as a continuous, evolving process. This process begins with diagnosis and embodied awareness, extends through the psychosocial negotiation of daily routines and relationships, and continues into interactions with healthcare systems and future-oriented identity work. Across these interconnected domains, participants actively constructed meaning, recalibrated priorities, and adapted practices, indicating that self-management is not merely behavioural but deeply interpretive, relational, and embedded within broader life contexts.

While previous research has documented the psychosocial burden of T2DM [47,50], this study extends existing literature by demonstrating that participants were not passive recipients of medical advice but active agents who continuously interpreted, adapted, and, at times, resisted prescribed management practices. Self-management emerged as a context-dependent and meaning-laden process, shaped by emotional states, social expectations, and competing life demands.

Living with T2DM presented ongoing challenges, particularly in sustaining lifestyle changes and managing the emotional burden of self-regulation. While previous research has documented the impact of stress and low mood on adherence [51,52], our findings show that these challenges were actively interpreted and integrated within the broader context of participants’ lives. Participants negotiated medication use, lifestyle adjustments, and monitoring in relation to personal meaning, social roles, and daily routines, demonstrating that self-management is not a series of discrete behaviours but an ongoing interpretive process. Emotional states, competing priorities, and access to resources shaped how practices were enacted, reflecting the complex interplay of individual, relational, and situational factors. By situating participants’ experiences within a framework of humanised care, relational support, and patient agency, the present findings demonstrate that self-management is actively co-constructed across social, emotional, and technological contexts [49,53,54]. While prior research has emphasised the role of empathy and relational support in shaping patient engagement [49], as well as the capacity of digital tools to enhance self-efficacy, connectedness, and adherence [53,54], this study advances these perspectives by showing how these elements are not experienced in isolation. Instead, midlife women with T2DM dynamically integrate humanised care and technology-mediated support into their everyday lives, negotiating these resources in relation to their evolving identities, social roles, and lived realities. In doing so, the findings position chronic disease management as a relational and interpretive process, highlighting how care practices are continuously shaped, adapted, and reconfigured within the complexity of daily life.

A key contribution of this study is the identification of ongoing identity work as central to living with T2DM in midlife. Participants actively negotiated how they understood themselves in relation to the condition, often attempting to maintain continuity with pre-diagnosis identities while incorporating new health-related practices. In some cases, this involved reframing the condition in less medicalised terms, suggesting that identity preservation and reconstruction are integral to sustained engagement with self-management.

Psychosocial dimensions were central to participants’ experiences. Dietary practices, symptom management, and daily routines intersected with relationships, social participation, and cultural expectations [55]. Rather than passively experiencing these challenges, participants actively negotiated social norms, dietary restrictions, and identity labels, maintaining a sense of continuity while adapting to the demands of T2DM. Our study extends previous work by showing that psychosocial engagement is intrinsically linked to self-management and humanised care [49,56], with emotional well-being and relational support shaping how participants enacted daily disease management.

Digital platforms and online communities were described not only as practical tools for monitoring and adherence but also as spaces where participants accessed emotional support, social validation, and experiential knowledge, aligning with existing evidence on the role of support systems in chronic illness management [57]. Importantly, our findings suggest that these platforms function as extensions of humanised care rather than merely supplementary resources. Through online engagement, participants were able to access forms of empathy, shared understanding, and relational connection that were not always available within formal healthcare settings. This highlights how digital environments can operationalise key principles of humanised care – such as recognition, responsiveness, and person-centred support- within everyday contexts [49]. Consistent with previous research on technology-mediated care [53,54], digital tools were perceived as enhancing self-efficacy and connectedness; however, our study extends these insights by demonstrating how midlife women actively integrate these resources into their identity work and daily self-management practices. Instead of passively consuming information, participants selectively engaged with online content to make sense of their condition, normalise their experiences, and negotiate their roles within social and familial contexts. In this way, digital platforms became embedded within the broader interpretive process of living with T2DM, supporting not only behavioural regulation but also meaning-making, identity negotiation, and emotional adaptation.

The findings also highlight the central role of patient agency within structurally and relationally constrained contexts. Participants described variable experiences with healthcare provision, with some reporting supportive and continuous care, while others experienced gaps that required them to assume greater responsibility for managing their condition [58,59]. In response, women engaged in self-directed learning, independent information-seeking, and the use of digital resources. This suggests that self-management is co-constructed across formal healthcare systems and informal or self-initiated practices.

Participants expressed concerns about long-term complications, consistent with research on illness perceptions in diabetes [60]. These concerns were not only experienced as sources of distress but also functioned as actively mobilised interpretive resources. Women drew on anticipated risks to guide reflection, recalibrate behaviours, and negotiate their sense of self in relation to the condition. In this way, diabetes management appears not solely driven by risk awareness, but constituted through an ongoing process of meaning-making situated within social, emotional, and relational contexts. This perspective positions future-oriented concerns as integral to identity work and adaptive self-management, foregrounding their active role in shaping engagement with the condition.

Importantly, the study also highlights that living with T2DM involves both constraint and potential for adaptation and growth. While participants described challenges and uncertainty, some also reported increased self-awareness, redefined priorities, and a more holistic understanding of well-being. These findings extend existing literature on adaptation and post-traumatic growth [61] by illustrating that processes of growth are embedded within everyday self-management practices, rather than occurring only in response to major life disruptions.

### Contradictory or unexpected findings

The study revealed important variations in how women experienced and interpreted living with T2DM. While some participants described distress, disruption, and uncertainty, others reported increased awareness, empowerment, and positive lifestyle change. These findings challenge deficit-focused narratives of chronic illness by demonstrating that, in some cases, diagnosis can act as a catalyst for reflection, learning, and proactive health behaviours [62]. Notably, the duration since diagnosis did not consistently predict adaptation or self-management success, suggesting that individual experiences are shaped by broader psychosocial and contextual factors rather than time alone.

Participants also highlighted the evolving role of digital networks. While previous research has noted the value of online communities in chronic disease management [63], our findings show that some women even prefer digital communities over immediate family for support, suggesting that virtual spaces can provide meaningful guidance, social validation, and identity affirmation, sometimes more effectively than traditional support networks. Cultural adaptation further illustrated the dynamic nature of living with T2DM. Some women described creatively modifying, rather than abandoning, cultural dietary practices, reinforcing their cultural identity while adhering to diabetes management goals. This challenges the assumption that effective self-management requires disengagement from traditional norms and suggests that culturally sensitive dietary counselling can enhance both adherence and patient satisfaction. Interestingly, negative experiences with healthcare did not uniformly lead to disengagement. Several participants responded proactively, seeking additional knowledge, experimenting with lifestyle modifications, and engaging with digital resources. This highlights patient agency and resilience, demonstrating that suboptimal care can catalyse active self-management rather than passive withdrawal. These findings extend previous research suggesting that negative healthcare experiences typically reduce engagement [64], by showing that individual motivations, social support, and access to digital resources can mediate responses to healthcare challenges.

Moreover, the emotional impact of diabetes was more heterogeneous than anticipated. While some women reported stress, isolation, or frustration, others described experiences of empowerment, increased self-awareness, and post-traumatic growth [61]. This variability indicates that emotional responses to T2DM are complex and context-dependent, and that living with the condition can catalyse unexpected positive adaptations in personal identity, lifestyle, and coping strategies.

Together, these insights underscore the unique contribution of our study: midlife women actively navigate the interplay of emotional, social, and practical dimensions of diabetes, revealing patterns of resilience and adaptation that go beyond traditional deficit-focused narratives of chronic illness.

### Limitations

Despite efforts to recruit women from diverse ethnic backgrounds, the final sample remained relatively homogeneous and relatively small, which may limit the transferability of the findings. In qualitative research, transferability is achieved through the provision of rich contextual detail rather than statistical representativeness; nonetheless, the limited size and diversity of our sample may constrain the extent to which findings are applicable to broader or more heterogeneous populations.

Several potential sources of bias should be considered. Self-selection bias is likely, as participants were recruited through social media and a pre-existing research engagement database. Individuals who chose to participate may have had higher health literacy, greater digital engagement, or a stronger interest in diabetes, which may have influenced the perspectives captured. This recruitment approach, while enabling wider geographical reach and flexible participation, may have shaped the nature of the data by privileging more engaged or digitally confident individuals. Relatedly, digital recruitment may have introduced access and literacy bias, potentially underrepresenting individuals with limited access to technology or lower digital confidence. As a result, the findings may not fully reflect the experiences of less digitally connected populations, which should be considered when interpreting transferability.

Recall bias may have influenced participants’ accounts, particularly for those with longer durations since diagnosis. In addition, self-reported narratives may be shaped by social desirability, with participants presenting their experiences in ways that align with perceived expectations.

The exclusion of individuals with diagnosed mental health conditions, including depression and anxiety, represents an important limitation. While intended to reduce confounding influences, this decision may have limited exploration of psychosocial burden, which is central to the experience of living with T2DM. As such, the findings may not fully capture the range of emotional and psychological experiences associated with the condition.

Finally, although the inclusion criterion specified a minimum diagnosis duration of two years, a small number of participants had been diagnosed for only one year. This deviation may have influenced the range of experiences reported, particularly regarding adaptation and self-management strategies, and should be considered when interpreting the findings.

### Strengths

This study provides rich, in-depth insights into the experiences of midlife women living with T2DM, a population that has been relatively underexplored in qualitative research on T2DM. By using semi-structured interviews, participants were able to share detailed personal accounts, capturing the complexities of daily management, emotional experiences, and identity negotiation that are often not accessible through quantitative approaches. Focusing specifically on midlife women allowed the study to illuminate experiences at a distinct life stage, addressing gaps in the literature regarding age-, gender-, and context-specific aspects of chronic illness management. The combination of rich qualitative data, interpretive thematic analysis, and attention to psychosocial and technological factors enhances the study’s contribution, framing T2DM as a complex, context-sensitive, and socially embedded condition.

The application of reflexive thematic analysis facilitated a rigorous interpretive approach, allowing the exploration of meaning-making, personal growth, and psychosocial processes beyond surface-level description. This approach highlights not only what women experience, but how they actively construct understanding and agency in managing T2DM.

Importantly, the recruitment strategy, while discussed as a limitation regarding potential selection bias, also allowed for a broader geographical reach and flexible participation, increasing the inclusivity of perspectives from women who might otherwise have been unable to participate. This approach enhanced the depth and diversity of experiential data, supporting a nuanced understanding of T2DM management in midlife.

### Practical implications

The findings underscore the need for more integrated and person-centred approaches to diabetes care that address both medical and psychosocial dimensions. Interventions should move beyond a sole focus on behavioural compliance to consider emotional well-being, identity, and social context.

Approaches such as cognitive behavioural therapy (CBT) and motivational interviewing (MI) may support emotional regulation and sustained behaviour change. Healthcare professionals should adopt collaborative models of care that recognise patients as active participants, supporting shared decision-making and personalised management strategies [65].

The findings also support the integration of digital platforms into care pathways, recognising their role in providing accessible information, peer support, and opportunities for self-directed learning. Incorporating these resources into formal care structures may enhance engagement and support more holistic management [66].

### Future research

Future research should explore how experiences of T2DM evolve over time through longitudinal designs, particularly in relation to identity, adaptation, and self-management practices. Cross-cultural studies are also needed to examine how social and cultural contexts shape experiences and management strategies. Further research should include individuals with common mental health comorbidities to better understand the intersection between psychological well-being and chronic disease management. In addition, the role of digital platforms in supporting long-term engagement and psychosocial outcomes warrants further investigation.

## Conclusion

This study that living with T2DM in midlife is a complex and evolving process shaped by the interplay of biological, psychological, social, and technological factors. Diabetes management extends beyond clinical control, involving ongoing adaptation, interpretation, and negotiation of identity, relationships, and daily life. A more holistic, person-centred approach to care is essential to support meaningful and sustainable management. Integrating psychosocial support, cultural sensitivity, and digital resources into diabetes care may enhance both clinical outcomes and quality of life for women living with T2DM.

## Supporting information

S1 Appendix COREQ checklist. (PDF)

S2 Appendix Interview schedule and topic guide. (DOCX)

S3 Appendix Expanded qualitative data matrix with thematic coding of participant interviews (N = 13). (DOCX)
