# Supplementary material for: A qualitative study of midlife women with type 2 diabetes in the UK: Exploring the impact of diabetes on their well-being
Source: PLoS One. 2026 Jun 1;21(6):e0350089. doi: 10.1371/journal.pone.0350089 (PMC13225358; doi:10.1371/journal.pone.0350089)
Supplement: S2 Appendix — (DOCX) [file pone.0350089.s002.docx]

**Appendix B: Interview Schedule and topic guide**

The interview schedule was developed through a six-step process:

1. Defining Objectives: The primary aim was to understand women's perspectives on T2DM, particularly focusing on psychosocial aspects and potential ethnic differences in perception and coping strategies.

2. Literature Review: An extensive review of existing literature was conducted to identify prevalent themes around diabetes and its impact on wellbeing. Key themes included diabetes-distress and its association with disease management, psychosocial aspects of the disease, the quality of care.

3. Question Generation: Based on identified themes, questions were brainstormed to facilitate discussion. These questions encompassed understanding of diabetes, daily life impacts, common themes such as diabetes-related distress, management, and ethnic differences in disease perception and management.

4. Feedback from Research Team: A preliminary set of questions was shared with the supervisor for feedback, ensuring comprehensiveness and relevance.

5. Refinement of Questions: Following supervisor’s feedback, the questions were refined for clarity and relevance, eliminating redundancy and ensuring alignment with the study's objectives.

6. Pilot Testing: The interview schedule was preliminarily tested within the research team and with a patient and then with one public involvement participant with type 2 diabetes to identify and rectify any issues with question clarity or phrasing.

Interview Topic Guide

 Please tell me your age

 How would you describe your ethnicity?

 How do you navigate the intersectionality of being a woman and having type 2 diabetes within your cultural community? For instance, specific societal expectations that influence how you manage your diabetes within your cultural community.

 How do you perceive the concept of wellbeing in relation to your experience with type 2 diabetes?

 How old were you when you were diagnosed with type 2 diabetes?

 Did you have concerning symptoms? Or having them now?

 A lot of people with diabetes describe their difficulties for their adaptation to the required lifestyle changes. Is this the case for you?

 How do you feel about these changes?

 Many people with diabetes find their emotions affect their diabetes. How have you found managing your diabetes? And has this impacted on your emotional wellbeing?

 How would you describe healthcare professional’s care? Do you find them willing to help you understand better the condition?

 Have you ever felt the need to hide diabetes from people?

 Have you ever faced any unusual behavior because of diabetes? What are some common misconceptions or stereotypes associated with diabetes in your culture?

 Has your diabetes ever affected your work, education or your relationships?

 Has social media ever played a role in helping you with your diabetes? Ie joining a group /gather information?

 Which is the most important source for you to gather information?

 Which are the sources of support during your ‘diabetes journey’? How about the social support?

 Have you ever tried to ‘build’ your coping strategies, for example, resilience through psychological support?

 What do the majority of people in your culture think about medical conditions and medications?
