# Supplementary material for: A qualitative study of midlife women with type 2 diabetes in the UK: Exploring the impact of diabetes on their well-being
Source: PLoS One. 2026 Jun 1;21(6):e0350089. doi: 10.1371/journal.pone.0350089 (PMC13225358; doi:10.1371/journal.pone.0350089)
Supplement: S3 Appendix — (DOCX) [file pone.0350089.s003.docx]

**S3 Appendix: Expanded qualitative data matrix with thematic coding of participant interviews (N = 13)**

### **THEME 1: Living with Type 2 Diabetes**

#### **1.1 Diagnosis and Health History | (Code: Diagnosis)**

| **Participant** | **Sub-theme** | **Expanded Quotes** | **Code** |
| --- | --- | --- | --- |
| P1 | Diagnosis & health history | “I was diagnosed several years ago… it came as a shock.” | Diagnosis |
| P2 | Diagnosis & health history | “It was picked up in routine blood tests. I had no concerning symptoms.” | Diagnosis |
| P3 | Diagnosis & health history | “I had been unwell for months before diagnosis.” | Diagnosis |
| P4 | Diagnosis & health history | “Diabetes runs in my family. I was diagnosed by myself… I suspect I had diabetes for a long time before I was diagnosed.” | Diagnosis |
| P5 | Diagnosis & health history | “Detected during pregnancy screening.” | Diagnosis |
| P6 | Diagnosis & health history | “It came up during a general health check.” | Diagnosis |
| P7 | Diagnosis & health history | “I was diagnosed after hospital admission.” | Diagnosis |
| P8 | Diagnosis & health history | “Found during unrelated medical tests.” | Diagnosis |
| P9 | Diagnosis & health history | “I was diagnosed with diabetes when I was 40 years old. It was a shock for me, as no one in my family had ever been diabetic.” | Diagnosis |
| P10 | Diagnosis & health history | “I’ve had diabetes for 23 years.” | Diagnosis |
| P11 | Diagnosis & health history | “Diagnosed about two years ago.” | Diagnosis |
| P12 | Diagnosis & health history | “Diagnosed during COVID hospitalisation.” | Diagnosis |
| P13 | Diagnosis & health history | “Detected during COVID admission; they took bloods and my HbA1c was off the charts.” | Diagnosis |

#### **1.2 Symptoms and Effects | (Code: Symptoms)**

| **Participant** | **Sub-theme** | **Expanded Quotes** | **Code** |
| --- | --- | --- | --- |
| P1 | Symptoms & effects | “I felt constantly tired and thirsty.” | Symptoms |
| P2 | Symptoms & effects | “I didn’t really notice anything at first.” | Symptoms |
| P3 | Symptoms & effects | “Weight gain and exhaustion were noticeable. My symptoms were severe before diagnosis… I was tired all the time, and my sugar levels were extremely high.” | Symptoms |
| P4 | Symptoms & effects | “Blurred vision and fatigue were my symptoms. I was ill for a long time before diagnosis… I have pain in my feet.” | Symptoms |
| P5 | Symptoms & effects | “High sugar levels were found unexpectedly. I was very tired and thirsty.” | Symptoms |
| P6 | Symptoms & effects | “I was gaining weight. No clear symptoms initially.” | Symptoms |
| P7 | Symptoms & effects | “Extreme fatigue and thirst.” | Symptoms |
| P8 | Symptoms & effects | “I had no symptoms at the time.” | Symptoms |
| P9 | Symptoms & effects | “Energy levels changed noticeably.” | Symptoms |
| P10 | Symptoms & effects | “Rapid weight gain, thirst, and urination.” | Symptoms |
| P11 | Symptoms & effects | “Lost weight and improved health markers.” | Symptoms |
| P12 | Symptoms & effects | “HbA1c was extremely high.” | Symptoms |
| P13 | Symptoms & effects | “I don’t have any symptoms; my eyes are fine, my feet are fine.” | Symptoms |

#### **1.3 Management Strategies | (Code: Management)**

| **Participant** | **Sub-theme** | **Expanded Quotes** | **Code** |
| --- | --- | --- | --- |
| P1 | Management strategies | “I changed my diet completely and started walking.” | Management |
| P2 | Management strategies | “I take medication and try to control food. I go to the gym; I do not drink much alcohol.” | Management |
| P3 | Management strategies | “Lifestyle changes helped stabilise things. Regular exercise and adaptation of eating habits.” | Management |
| P4 | Management strategies | “I had to change diet and exercise habits.” | Management |
| P5 | Management strategies | “Diet and monitoring became essential. I changed my diet… I started eating Greek yogurt and reduced carbs.” | Management |
| P6 | Management strategies | “Exercise and medication are key.” | Management |
| P7 | Management strategies | “I’ve tried to cut down carbs, I exercise, and I lost weight.” | Management |
| P8 | Management strategies | “I adjusted lifestyle gradually. I’ve stopped eating the same as my husband.” | Management |
| P9 | Management strategies | “I’m active. I go swimming. I go to the gym… I control my diet.” | Management |
| P10 | Management strategies | “Bariatric surgery and strict diet. I do a lot of mindfulness.” | Management |
| P11 | Management strategies | “Exercise and diet adherence.” | Management |
| P12 | Management strategies | “I do more exercise… I’ve lost weight.” | Management |
| P13 | Management strategies | “Diet adjustment and walking. It might just be 20 minutes per day; a lot of vegetables and fruits, less carbs; calming my mind… and meditation.” | Management |

#### **THEME 2: Psychosocial Dimensions**

#### **2.1 Psychological and Emotional Impact | (Code: Psychological Impact)**

| **Participant** | **Sub-theme** | **Expanded Quotes** | **Code** |
| --- | --- | --- | --- |
| P1 | Psychological and emotional impact | “It changed how I think about my health. It is very stressful… and because I am stressed, this affects my diabetes.” | Psychological Impact |
| P2 | Psychological and emotional impact | “I felt shocked when diagnosed. I am an emotional eater, so my emotions affect my diabetes.” | Psychological Impact |
| P3 | Psychological and emotional impact | “It was very stressful at the beginning. Diabetes makes you feel that you are unhealthy… I am an emotional eater; the way I feel affects my eating habits. When I am down, I eat unhealthy, and this affects my diabetes… This is stressful. At some point, diabetes made me feel hopelessness.” | Psychological Impact |
| P4 | Psychological and emotional impact | “Because of diabetes, my mental health is very poor… stress levels have made my diabetes worse… diabetes makes you feel useless and fatigued.” | Psychological Impact |
| P5 | Psychological and emotional impact | “I worry about future complications. Diagnosis made me feel disappointed and frustrated… diabetes makes me feel stressed.” | Psychological Impact |
| P6 | Psychological and emotional impact | “I try not to think about it too much. Diabetes has affected how I feel about myself… I am an emotional eater; when I am upset, I eat unhealthy, and this affects my symptoms.” | Psychological Impact |
| P7 | Psychological and emotional impact | “It affected my confidence. I have a condition I feel I shouldn’t have… diabetes made me feel angry.” | Psychological Impact |
| P8 | Psychological and emotional impact | “I am generally positive. I slowly adapted emotionally.” | Psychological Impact |
| P9 | Psychological and emotional impact | “Diabetes makes me feel up and down.” | Psychological Impact |
| P10 | Psychological and emotional impact | “Stress makes blood sugar worse… huge amounts of stress.” | Psychological Impact |
| P10 | Psychological and emotional impact | “Overeating can be self-harm.” | Psychological Impact |
| P11 | Psychological and emotional impact | “When I started the treatment, I was in panic.” | Psychological Impact |
| P12 | Psychological and emotional impact | “I sometimes eat emotionally. I was frightened and intimidated when I was first diagnosed.” | Psychological Impact |
| P13 | Psychological and emotional impact | “Cravings are emotional and hormonal.” | Psychological Impact |

#### **2.2 Social and Cultural Aspects | (Code: Social & Cultural Aspects)**

| **Participant** | **Sub-theme** | **Expanded Quotes** | **Code** |
| --- | --- | --- | --- |
| P1 | Social and cultural aspects | “I was diagnosed in Australia, both my parents had diabetes, and moving between countries like Poland and the UK made me aware that standards and perspectives on my sugar levels were different.” | Social & Cultural Aspects |
| P2 | Social and cultural aspects | “In England, they believe that if you have type 2 diabetes, it is because you eat the wrong things, you are not fit, and you are overweight.” | Social & Cultural Aspects |
| P3 | Social and cultural aspects | “People in India are not very comfortable with the idea of diabetes… diet and lifestyle are different there.” | Social & Cultural Aspects |
| P4 | Social and cultural aspects | “Family expectations affect me. People do not like fat people.” | Social & Cultural Aspects |
| P5 | Social and cultural aspects | “People believe that diabetes is your own responsibility.” | Social & Cultural Aspects |
| P7 | Social and cultural aspects | “Within my culture, diabetes is treated as something serious… sometimes people believe that I do not need support because I am fit… in my culture, some people believe that medication is necessary and others not.” | Social & Cultural Aspects |
| P8 | Social and cultural aspects | “It’s not only because of an unhealthy lifestyle.” | Social & Cultural Aspects |
| P9 | Social and cultural aspects | “Peers influence eating habits. A lot of other Irish people are diabetic… in my community, being diabetic is not something that is considered awful… some people believe that diabetic people are obese.” | Social & Cultural Aspects |
| P10 | Social and cultural aspects | “I don’t feel very British.” | Social & Cultural Aspects |
| P10 | Social and cultural aspects | “People giggled when my weight was read out.” | Social & Cultural Aspects |
| P11 | Social and cultural aspects | “Because I am half Chinese… food is a big part of the culture.” | Social & Cultural Aspects |
| P12 | Social and cultural aspects | “In my culture, people are very pleased to have access to medical help.” | Social & Cultural Aspects |
| P13 | Social and cultural aspects | “I’ve met people who trust everything doctors say… others who try to do research for themselves. I am not limited to one culture, as I live in London, which is multicultural.” | Social & Cultural Aspects |

#### **2.3 Daily Life Impact | (Code: Daily Life Impact)**

| **Participant** | **Sub-theme** | **Expanded Quotes** | **Code** |
| --- | --- | --- | --- |
| P1 | Daily life impact | “I changed my whole eating pattern. I have to leave my work.” | Daily Life Impact |
| P2 | Daily life impact | “Medication became part of daily life.” | Daily Life Impact |
| P3 | Daily life impact | “I had to plan meals carefully. I wasn’t socialising because I was afraid that my diet would be affected if I went out with friends… the stress because of diabetes affected my relationships.” | Daily Life Impact |
| P4 | Daily life impact | “Exercise became essential. Diabetes affected my educational opportunities and relationships.” | Daily Life Impact |
| P5 | Daily life impact | “Checking sugar levels daily.” | Daily Life Impact |
| P6 | Daily life impact | “Life became more structured.” | Daily Life Impact |
| P7 | Daily life impact | “Food choices became limited. I am not able to do things I used to do… diabetes made me fearful of big social events. I work in a university; sometimes after meetings, colleagues go for lunch together… I cannot eat what they eat.” | Daily Life Impact |
| P8 | Daily life impact | “Health monitoring is regular.” | Daily Life Impact |
| P9 | Daily life impact | “Social life changed slightly.” | Daily Life Impact |
| P10 | Daily life impact | “I had to stop working because of the symptoms and complications. I now have limited contact with my extended family.” | Daily Life Impact |
| P11 | Daily life impact | “Exercise increased significantly. Diabetes has affected my social life more than anything.” | Daily Life Impact |
| P12 | Daily life impact | “Sleep and rest became important.” | Daily Life Impact |
| P13 | Daily life impact | “Diabetes has affected my life, relationships, and work. I avoid some social events if I know there’s going to be food that I should avoid.” | Daily Life Impact |

## **THEME 3: Healthcare and Support**

## **3.1 Experiences with Healthcare System | (Code: Healthcare Experiences)**

| **Participant** | **Sub-theme** | **Expanded Quotes** | **Code** |
| --- | --- | --- | --- |
| P1 | Healthcare experience | “GP support was very good.” | Healthcare Experiences |
| P2 | Healthcare experience | “Most doctors are good and try to help. However, every time I see a different doctor, and this is difficult.” | Healthcare Experiences |
| P3 | Healthcare experience | “Mixed experiences with clinicians. The general nurse laughed when I asked to stop medication, then they referred me to a diabetes clinic. The doctors there were better and specialists.” | Healthcare Experiences |
| P4 | Healthcare experience | “I was telling doctors about my symptoms and they did not do anything. This was frustrating. They did not give me the right directions about lifestyle changes. Some doctors made me feel disliked.” | Healthcare Experiences |
| P5 | Healthcare experience | “Regular follow-ups helped, but it’s difficult to access the healthcare system. Sometimes professionals are not empathetic or compassionate.” | Healthcare Experiences |
| P6 | Healthcare experience | “They are good with routine checks. However, when I ask for a medication change, the answer is a flat no.” | Healthcare Experiences |
| P7 | Healthcare experience | “When I was diagnosed, a nurse told me diabetes is a lifestyle choice. This was a bad experience. However, generally the healthcare team is supportive.” | Healthcare Experiences |
| P8 | Healthcare experience | “The GP was not helpful. The quality of care is poor, although practice nurses try to be helpful.” | Healthcare Experiences |
| P9 | Healthcare experience | “The doctor really understands me. On the whole, I feel well looked after and I can ask my GP anything, although there are some gaps in support.” | Healthcare Experiences |
| P10 | Healthcare experience | “Sometimes care is judgemental. I have had both good and bad experiences. My new diabetic nurse is excellent and helps me understand my condition.” | Healthcare Experiences |
| P10 | Healthcare experience | “I had to self-manage and learn everything myself.” | Healthcare Experiences |
| P11 | Healthcare experience | “I was diagnosed late and I felt very angry with the healthcare system.” | Healthcare Experiences |
| P12 | Healthcare experience | “NHS care was very positive. I had an excellent GP and they gave me plenty of information.” | Healthcare Experiences |
| P13 | Healthcare experience | “Healthcare professionals are very supportive and understanding.” | Healthcare Experiences |

## **3.2 Information and Support Sources | (Code: Information Sources)**

| **Participant** | **Sub-theme** | **Expanded Quotes** | **Code** |
| --- | --- | --- | --- |
| P1 | Information sources | “I did research and used online programmes to learn more.” | Information Sources |
| P2 | Information sources | “Online resources are useful. Diabetes.co.uk is very helpful.” | Information Sources |
| P3 | Information sources | “Reading online studies, books and research is very helpful.” | Information Sources |
| P4 | Information sources | “NHS website is helpful.” | Information Sources |
| P5 | Information sources | “The internet is the most important source of information.” | Information Sources |
| P6 | Information sources | “Diabetes UK is very helpful. I also use social media and online groups.” | Information Sources |
| P7 | Information sources | “I am lucky to have access to the internet. I read about diabetes and its management. Diabetes UK is useful.” | Information Sources |
| P8 | Information sources | “I use online articles. I also searched online about medication.” | Information Sources |
| P9 | Information sources | “I use Diabetes UK and the internet. I also receive a magazine.” | Information Sources |
| P10 | Information sources | “For me, the Diabetes UK forum is useful, as well as some Facebook pages.” | Information Sources |
| P11 | Information sources | “Diabetes.co.uk is helpful.” | Information Sources |
| P12 | Information sources | “I find online websites helpful. I also look for recipes.” | Information Sources |
| P13 | Information sources | “NHS and YouTube are helpful.” | Information Sources |
| P13 | Information sources | “YouTube videos and podcasts were useful for understanding diabetes management. I have watched webinars and used the Diabetes UK website, NHS website, articles and books.” | Information Sources |

## **3.3 Challenges in Diabetes Management | (Code: Challenges)**

| **Participant** | **Sub-theme** | **Expanded Quotes** | **Code** |
| --- | --- | --- | --- |
| P1 | Challenges in management | “It was difficult to adjust my habits at first. It is difficult to give so much time to your health. I struggle with keeping my diet and my last results are not great.” | Challenges |
| P2 | Challenges in management | “Controlling what I eat is not always easy. Adapting your lifestyle is quite hard. I love chocolate and I’ve gained weight. Alongside other health issues, diabetes is an additional problem.” | Challenges |
| P3 | Challenges in management | “It took time to find a routine that works. Feeling hungry all the time is difficult.” | Challenges |
| P4 | Challenges in management | “Nobody told me what changes I should make to my lifestyle. It is too difficult to manage all these symptoms.” | Challenges |
| P5 | Challenges in management | “Monitoring and diet require constant attention. Comorbid conditions make diabetes management more difficult.” | Challenges |
| P6 | Challenges in management | “Staying consistent is challenging. I find it difficult to balance lifestyle changes.” | Challenges |
| P7 | Challenges in management | “Strict control of food is difficult to maintain.” | Challenges |
| P9 | Challenges in management | “It can be hard to balance everything. Menopause was very difficult for me because of diabetes.” | Challenges |
| P10 | Challenges in management | “Keeping weight loss is an ongoing struggle.” | Challenges |
| P11 | Challenges in management | “Keeping it up is the challenge. Diabetes management is extreme and managing eating is challenging for me.” | Challenges |
| P12 | Challenges in management | “Adapting to all the changes was not easy. It is challenging to be disciplined.” | Challenges |
| P13 | Challenges in management | “I haven’t had an easy time. I have sugar and carbohydrate cravings.” | Challenges |

## **THEME 4: Outlook and Personal Development**

## **4.1 Attitudes Towards Treatment | (Code: Treatment Attitudes)**

| **Participant** | **Sub-theme** | **Expanded Quotes** | **Code** |
| --- | --- | --- | --- |
| P1 | Attitudes towards treatment | “I try to stay positive.” | Treatment Attitudes |
| P2 | Attitudes towards treatment | “It’s about keeping a balance. Regular checks are useful.” | Treatment Attitudes |
| P3 | Attitudes towards treatment | “When I was first diagnosed, I was very adamant and I didn’t want to go on medication. Doctors seemed to want me to be on medication.” | Treatment Attitudes |
| P5 | Attitudes towards treatment | “Tablets are not helpful. You need to change your lifestyle.” | Treatment Attitudes |
| P7 | Attitudes towards treatment | “Sometimes tablets do not make any difference to your body. I try to balance everything. There is no one treatment which works for everyone. Treatments should be individualised.” | Treatment Attitudes |
| P8 | Attitudes towards treatment | “Treatment is a balance of mental and physical health. It is about finding a lifestyle which is acceptable and pleasurable for you.” | Treatment Attitudes |
| P9 | Attitudes towards treatment | “Medication is an important part of diabetes treatment.” | Treatment Attitudes |
| P10 | Attitudes towards treatment | “It’s part of who I am now. Treatment is about blood sugar control and minimising the risk of further complications. Treatment is a balance of being physically and mentally well.” | Treatment Attitudes |
| P11 | Attitudes towards treatment | “Medication is an important part of diabetes treatment.” | Treatment Attitudes |
| P13 | Attitudes towards treatment | “I do not like saying I have type 2 diabetes or identifying as diabetic. I try to support my metabolism and think of myself as a healthy eater rather than someone with a disease.” | Treatment Attitudes |

## **4.2 Long-term Concerns | (Code: Long-term Concerns)**

| **Participant** | **Sub-theme** | **Expanded Quotes** | **Code** |
| --- | --- | --- | --- |
| P2 | Long-term concerns | “I worry about complications. My diabetes may get worse, and I will have to keep lifestyle changes for the rest of my life.” | Long-term Concerns |
| P4 | Long-term concerns | “My symptoms are getting worse and worse, and I will feel completely exhausted.” | Long-term Concerns |
| P5 | Long-term concerns | “Fear of progression.” | Long-term Concerns |
| P6 | Long-term concerns | “My diabetes may become uncontrollable and I could end up in hospital.” | Long-term Concerns |
| P7 | Long-term concerns | “I’ve got a life ahead of me. I fear diabetes complications and long-term consequences.” | Long-term Concerns |
| P9 | Long-term concerns | “I fear that because of diabetes I will have a short life.” | Long-term Concerns |
| P10 | Long-term concerns | “I fear long-term complications.” | Long-term Concerns |
| P11 | Long-term concerns | “I was diagnosed late and I fear a worse prognosis.” | Long-term Concerns |
| P13 | Long-term concerns | “I don’t want complications or lifelong medication. I worry that diabetes will get worse and cause further blood sugar imbalances.” | Long-term Concerns |

## **4.3 Positive Aspects and Opportunities | (Code: Positive Aspects)**

| **Participant** | **Sub-theme** | **Expanded Quotes** | **Code** |
| --- | --- | --- | --- |
| P1 | Positive aspects and opportunities | “My diagnosis made me more aware of my family, and I now offer more support to my mum.” | PositiveAspects |
| P2 | Positive aspects and opportunities | “When you are on a diet, you can see a point and achieve a more balanced lifestyle.” | PositiveAspects |
| P3 | Positive aspects and opportunities | “Because of diabetes I started searching and reading about how I can improve my lifestyle. My goal is to put diabetes into remission.” | PositiveAspects |
| P4 | Positive aspects and opportunities | “I searched for information online myself to better understand my condition.” | PositiveAspects |
| P5 | Positive aspects and opportunities | “I volunteered and did research to help others and better understand my condition.” | PositiveAspects |
| P6 | Positive aspects and opportunities | “At first I was distressed and socially withdrawn, but later I had the opportunity to better understand my condition and learn from other people with diabetes.” | PositiveAspects |
| P7 | Positive aspects and opportunities | “I had the opportunity to change my mindset in general. I realised I had to make lifestyle changes, which improved my life.” | PositiveAspects |
| P8 | Positive aspects and opportunities | “Health habits improved.” | PositiveAspects |
| P9 | Positive aspects and opportunities | “I’ve improved my life and I am calmer now.” | PositiveAspects |
| P10 | Positive aspects and opportunities | “Having diabetes motivated me to take part in research to help others better understand lived experiences of the condition, which I find rewarding. I also volunteered for Diabetes UK.” | PositiveAspects |
| P11 | Positive aspects and opportunities | “Dealing with diabetes has helped me improve my overall wellbeing. I also had the opportunity to join a diabetes association.” | PositiveAspects |
| P13 | Positive aspects and opportunities | “I find it beneficial. I reflect more, I am motivated, and I have improved my habits and lifestyle.” | PositiveAspects |

## **4.4 Personal Growth and Self-awareness | (Code: Personal Growth)**

| **Participant** | **Sub-theme** | **Expanded Quotes** | **Code** |
| --- | --- | --- | --- |
| P1 | Personal growth and self-awareness | “I am more motivated to change. With the research I did, I increased my self-awareness.” | PersonalGrowth |
| P2 | Personal growth and self-awareness | “It is a journey. You learn a lot, you do your own research and you become stronger.” | PersonalGrowth |
| P3 | Personal growth and self-awareness | “I learned to manage better. Diabetes is a lonely journey; you have to rely on yourself and find information.” | PersonalGrowth |
| P4 | Personal growth and self-awareness | “I became more health conscious.” | PersonalGrowth |
| P5 | Personal growth and self-awareness | “I don’t fit into anyone’s box. I found what works for me. I built my coping strategies and resilience.” | PersonalGrowth |
| P6 | Personal growth and self-awareness | “More disciplined lifestyle now. I became more patient and resilient.” | PersonalGrowth |
| P7 | Personal growth and self-awareness | “Diabetes helped me realise that life is too short. I decided to test what works best for me. I gathered information on my own, went back to doctors and asked for individualised support.” | PersonalGrowth |
| P8 | Personal growth and self-awareness | “I am resilient. I fail every single day, but this does not bother me.” | PersonalGrowth |
| P9 | Personal growth and self-awareness | “More conscious of health. I was confused about how to use the glucose monitor, so I searched online for instructions.” | PersonalGrowth |
| P10 | Personal growth and self-awareness | “When I was first diagnosed I almost grieved for my old life. However, I did not give up. I tried my best and learned a lot from this journey.” | PersonalGrowth |
| P11 | Personal growth and self-awareness | “It made me reflect more on life. I did my own investigation.” | PersonalGrowth |
| P12 | Personal growth and self-awareness | “Wellbeing is calmness and balance. I’ve learned to reflect on my experiences and emotions.” | PersonalGrowth |
| P13 | Personal growth and self-awareness | “Better understanding of metabolism. I’ve become more careful and self-aware, and mindful of my stress levels.” | PersonalGrowth |
